# Pro-Environmental Behavior Research: Theoretical Progress and Future Directions

**DOI:** 10.3390/ijerph19116721

**Published:** 2022-05-31

**Authors:** Hong Tian, Xinyu Liu

**Affiliations:** School of Business and Management, Jilin University, Changchun 130012, China; xinyu20@mails.jlu.edu.cn

**Keywords:** pro-environmental behavior, literature research, theoretical progress, future direction

## Abstract

Realistic environmental problems drive the growth of pro-environment behavior research, among which the most important progress is about the theoretical innovation and development of pro-environmental behavior. Thus, the main purpose of this paper was to review the literature and help researchers to understand the theoretical progress of pro-environmental behavior. This study systematically analyzed 1806 papers published in SCI-EXPANDED and SSCI databases. It presented the research overview of pro-environmental behavior in terms of status of literature publication, research hotspots and topics. On this basis, this paper further focused on key theoretical papers and summarized three paths of theoretical progress for pro-environmental behavior: theoretical development, theoretical exploration and theoretical integration. Along the theoretical development path, studies mainly apply theories of psychology, sociology and economics to analyze and explain the formation and consequences of pro-environmental behavior. In terms of theoretical exploration, existing studies propose and develop value-belief-norm theory, behavioral theories related to contexts and pro-environmental behavior decision models. Theoretical integration is the direction of future research, such as the combination of rationality and sensibility, and the combination of external and internal causes. Therefore, this paper summarized the theoretical progress of pro-environmental behavior and proposed future research directions, which contribute to its theoretical development.

## 1. Introduction

In recent years, climate challenges have intensified, posing a threat to human existence and life [1]. According to UN Climate Change News, in 2020 alone, climate shocks forced 30 million people to flee their homes. In 2021 alone, extreme weather caused about USD 120 billion in insured losses [2]. With the prominence of various environmental problems, how to deal with these problems and improve environmental sustainability has attracted extensive attention [3,4]. In essence, environmental problems stem from human behavior [5]. The change of behavior is a necessary condition to improve the environmental situation. Pro-environmental behavior plays an important role in decreasing the waste of natural resources, reducing the emission level of pollutants and weakening environmental damage, thus being an effective way to deal with environmental problems and protect environmental sustainability [6,7]. Therefore, the study of pro-environmental behavior has become one of the fields with both research value and research prospects, and has attracted the attention of scholars from many disciplines.

Throughout the existing literature, the research content of pro-environmental behavior is widely distributed. Based on the different roles such as students [8,9], employees [10], tourists [11,12], and residents [13,14,15], scholars have conducted researches on the pro-environmental behavior of individuals, which include its conceptual connotation, formation mechanism, effect pathway, and so on. In order to promote the systematization of pro-environmental behavior knowledge and better guide the follow-up research, some scholars have summarized and prospected the research on pro-environmental behavior from different perspectives, mainly focusing on the following aspects. Firstly, the summary and explanation of the influencing factors or results of pro-environmental behavior [16,17,18], which accounts for a considerable proportion in the reviews. Secondly, the relationship between pro-environmental behavior and other research topics were discussed, such as social norms and pro-environmental behavior [19], and pro-environmental behavior and subjective well-being [20]. Thirdly, the theoretical model of pro-environmental behavior was constructed, such as the conceptual model for voluntary pro-environmental behavior of employees [21]. These literatures are of great significance to systematically understand the existing research on pro-environmental behavior. However, current academic research on pro-environmental behavior is in a vigorous development stage. The antecedents, consequences, and important theoretical basis of the behavior are being explored constantly, and no consistent conclusion has been reached [3]. Among them, the development of theory, as the cornerstone of in-depth research and sustainable development of the field, has attracted much attention in recent years. Unfortunately, there is a lack of literature summarizing and discussing the theoretical progress of pro-environmental behavior, which is not without regret for the further development of pro-environmental behavior research. Therefore, this paper is devoted to reviewing the literature, focusing on the theoretical progress of pro-environment behavior, and providing a theoretical scan for subsequent scholars.

This paper analyzed the existing literature on pro-environmental behavior and summarized its research status. After that, this study focused on the key theoretical literatures. Through in-depth reading and content mining of them, a framework for the theoretical progress of pro-environmental behavior was proposed. Through a comprehensive review and focused study of pro-environmental behavior literatures, this paper describes the research overview of pro-environmental behavior, clearly presents the theoretical progress of pro-environmental behavior, and proposes possible future research directions. It is expected to deepen the understanding of the research status and theoretical basis of pro-environmental behavior among scholars interested in this research issue, and provide theories and guidance for their future research, which may make contributions to the theoretical development of pro-environmental behavior. In addition, governments and managers can also deepen their understanding of the behavior, which is helpful in formulating appropriate policies to improve individual environmental behaviors.

## 2. Definition of Pro-Environmental Behavior

There is no obvious disagreement or debate among different scholars on the definition of pro-environment behavior. The definition proposed earlier and widely cited is the behavior that minimizes the negative impact of one’s own behavior on the environment [22], emphasizing the autonomy of actors and reducing harm to the world. Further, based on the perspective of the impact of behavior on the environment, the definition of pro-environmental behavior is subsequently extended to minimize the harm to the environment and even benefit it [23]. This definition focuses on improving environmental conditions while paying attention to reducing negative impacts on the environment, which include greenhouse gas emissions, waste of natural resources, and so on. In addition, from the perspective of sustainability, pro-environmental behavior refers to behaviors that help to improve environmental sustainability [7]. On the whole, this paper argues that pro-environmental behavior refers to behavior that consciously protects the environment and improves its sustainability.

Based on the above definitions, there are many labels similar to pro-environmental behavior in the literature, such as “ecological behavior”, “environmental behavior”, “environmental action”, “responsible environmental behavior”, “ecological responsible behavior”, “conservation behavior”, “environmentally responsible behavior”, “environmentally significant behavior”, “pro-ecological behaviors”, “environmentally conscious behavior”, “environmentally friendly behavior”, “sustainable behavior”, “eco-friendly behavior”, and “green behavior”. The appearance of many different construct labels is largely unintentional, and they have some common ground in many aspects [24]. For example, they include the same specific behaviors such as reducing resource use. They are assumed to be affected by factors such as values, and are explained by theories such as the theory of planned behavior. Therefore, this paper has no intention to distinguish them obviously, and relevant literatures are included in the research scope.

## 3. Research Status

### 3.1. Data Sources, Analysis Tools, and Methods

Data sources should ensure the comprehensiveness of the scope of the paper [25], and the academic, relevance, and readability of the content of the paper. Therefore, this paper used the 15 terms mentioned above as topic keywords to search for papers related to pro-environmental behavior in SCI-EXPANDED and SSCI databases. A total of 6092 results were obtained (the date of data retrieval was 1 March 2022). By limiting literature types, only the peer-reviewed “article” and “review” papers were retained, resulting in 5937 records. Through manual check and screening of papers one by one, the irrelevant papers focusing on biology, chemistry, architecture, and other fields were removed, and papers not mainly studying the pro-environmental behavior at the individual level were deleted, resulting in 1806 literatures (shown in Table S1). 

Considering the comprehensive function of Citespace, this paper selected Citespace5.8R3 (Chaomei Chen, Drexel University, Philadelphia, PA, USA), the latest version of this software, as a tool for bibliometric analysis. Specifically, in terms of data extraction, Citespace5.8R3 could refine the data in the time slice as required, and the length of the time slice could be manipulated [25]. For example, the time slice was set to one year, and the selection criteria was set to select the top 100 levels of the most occurred items from each slice. In terms of analysis functions, Citespace5.8R3 could perform frequency statistics, co-occurrence analysis, cluster analysis, etc. Therefore, this software was used to perform a quantitative analysis on selected literatures to reveal the published status, research hotspots, and topics of the existing research on pro-environmental behavior.

In terms of analysis methods, frequency statistics refers to the calculation of the number of occurrences of the data to derive their frequency. Co-occurrence analysis mainly focuses on keywords and analyzes the co-occurrence of different keywords in the literature. Based on this, cluster analysis allows for the aggregation of closely linked keywords into clusters, with different clusters corresponding to different research topics.

### 3.2. Status of Literature Publication

**Figure 1:**
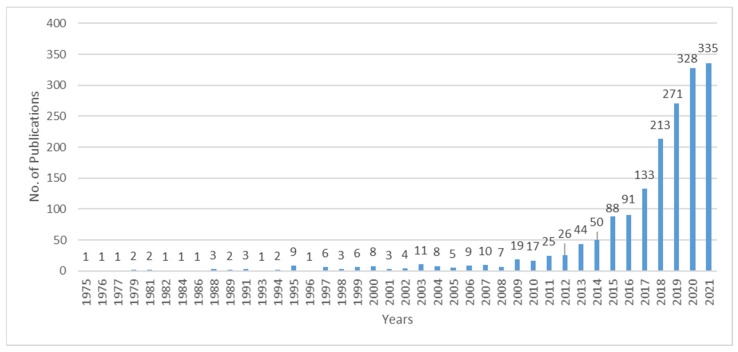
Time distribution of the number of published papers.

**Figure 2:**
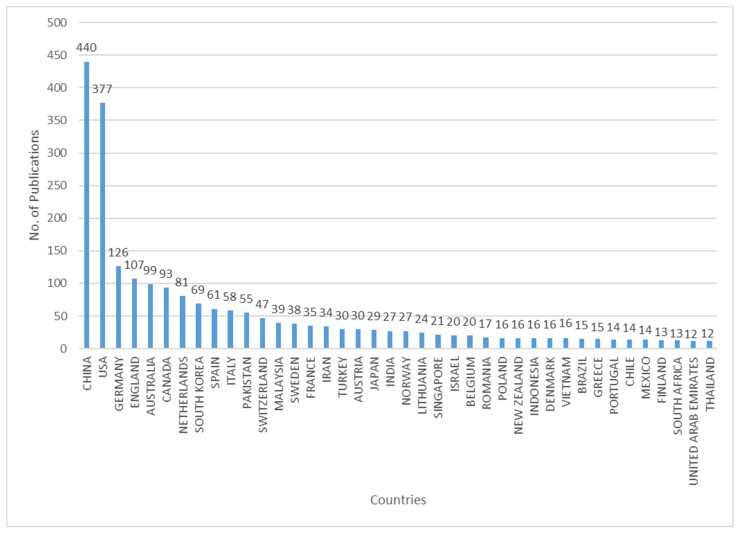
Geographical distribution of published papers.

Through statistical analysis of the selected 1806 literatures, the histograms depicting annual number and geographical distribution of literatures on pro-environmental behavior were obtained (Figure 1 and Figure 2). According to Figure 1, the earliest research on pro-environmental behavior in SCI-EXPANDED and SSCI databases was published in 1975. After that, the number of papers were few and fluctuated little for a long time. Since 2009, more than 10 papers have been published annually, indicating that pro-environmental behavior has become a stable research field. Since 2012, the number of documents has increased significantly, which suggests that pro-environmental behavior has gradually become a research hotspot. In the past two years, there has been a peak of publishing, with an annual volume of more than 300 papers. Scholars have become widely concerned with this research field. Figure 2 shows the countries with more than 10 publications. As can be seen from Figure 2, pro-environmental behavior research is widely distributed and involves many countries. 

### 3.3. Research Hotspots and Topic Analysis

Keywords are highly summarized and condensed contents of the literature. Taking them as the representative characteristics of the literature for co-occurrence analysis and cluster analysis can easily and accurately acquire the research hotspots and topics [3]. Specifically, Table 1 is the result of co-occurrence analysis, which reports the top ten keywords in the field of pro-environmental behavior research along with their frequency, centrality, and year of earliest occurrence. Among them, a higher frequency means more literature with the term as a keyword, which indicates that the relevant research issue is a research hotspot and scholars pay high attention to it. Centrality indicates the importance of the keyword, i.e., the degree of connection with other keywords. Table 2 shows the results of cluster analysis, presenting the top ten branch topics of pro-environment behavior research, which are summarized by Citespace5.8R3 based on log-likelihood ratio (LLR). Specifically, size indicates the number of members in a cluster, and silhouette describes the closeness or homogeneity between members within the cluster. Mean (year) represents the average year of the literature involved in the cluster. In view of the fact that research hotspots and topics are both obtained based on keywords and there are a lot of overlapped content between them, this paper makes an integrated exposition of them in combination with the table content.

According to Table 2, the research topics of pro-environmental behavior are mainly divided into three categories. The first type includes pro-environmental behavior studies focusing on different roles of individuals (#1, #2, #3, #5, #6). Typical roles are employees, tourists, residents, students, and consumers. For example, it has been shown that green human resource management or corporate social responsibility promotes employees to engage in pro-environmental behavior by improving organizational identity [26,27]. Place attachment was found to be an important reason for tourists to protect their destinations [28]. Environmental education could be an effective measure to encourage students to take green actions [29], because it provides necessary environmental protection knowledge. The second type focuses on key influencing factors and related theories to study the antecedents of pro-environmental behavior (#4, #7). Typical influencing factors include attitudes, norms, perceived behavior control, habits, values, beliefs, and so on. Among them, the effects of attitudes and values are research hotspots [30,31]. Corresponding theoretical bases are the theory of planned behavior and value-belief-norm theory. The third category is the research on the consequences of pro-environment behavior (#8, #9, #10). Happiness is a direct psychological result [20], which is also a psychological result that attracts much attention of scholars. In addition, pro-environmental behaviors also have spillover effects, including positive spillover effects based on efficacy or identity, and negative spillover effects represented by moral licensing effect [32].

## 4. Theoretical Progress of Pro-Environmental Behavior

**Figure 3:**
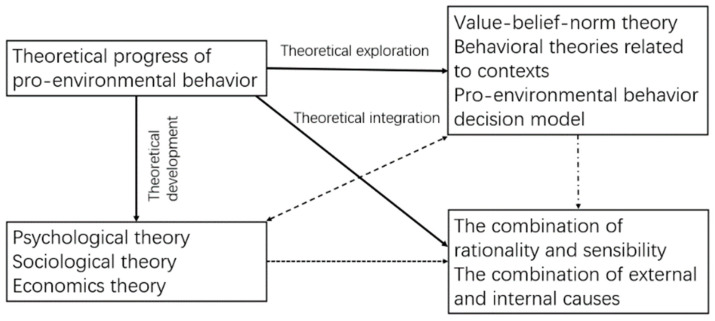
Theoretical progress of pro-environmental behavior.

Around the important research topics of pro-environmental behavior, that is, pro-environmental behavior of individuals with different roles, antecedents, and consequences of the behavior, relevant theories are developing continuously. This section focuses on the theoretical literature of pro-environmental behavior to review the evolution progress of theories related to pro-environmental behavior, and explores the evolution trend of them. Referring to the idea that innovation theory divides innovation into exploitative innovation and exploratory innovation [33,34], this paper roughly divides theoretical progress of pro-environmental behavior into theoretical development and theoretical exploration. The former refers to applying the existing theories from psychology, sociology and economics to explain the phenomena of pro-environmental behavior, which includes formation mechanism, spillover effect, and so on. The latter emphasizes the expansion of theories, mainly including two ways. One is to expand or supplement existing theories with pro-environmental behavior as the background. The other is to construct unique theoretical models based on the characteristics of pro-environmental behavior. On this basis, this paper regards theoretical integration as an important trend of theoretical evolution in the future. The interpretation of pro-environmental behavior by a single theory is usually limited [35]. A feasible method is to integrate existing theories to some extent to form complementary explanations, which could help improve the explanatory power of theories to practical situations. The overall framework of theoretical progress is shown in Figure 3. The theories involved are shown in Table 3.

### 4.1. Theoretical Development of Pro-Environment Behavior

#### 4.1.1. Application of Psychological Theories in the Study of Pro-Environmental Behavior

Early studies on pro-environmental behavior mainly focus on its influencing factors, and are relatively scattered. They generally explored the predictive effect of one or several variables, and did not put forward a systematic theoretical framework to explain the reasons for individuals to carry out the behavior [36,37]. The development of psychological theories provides references for pro-environmental behavior studies, which begin to involve the internal psychological process of individuals before taking the behavior. 

Among the psychological theories applied to the study of pro-environmental behavior, the most representative ones are the theory of planned behavior and norm activation theory [38]. As the successor of theory of reasoned action, the theory of planned behavior performs as an important theoretical basis to explain pro-environmental behavior. Moreover, it is also a hot content in the research [3,39,40]. Based on this theoretical perspective, scholars have investigated the impact of attitudes, subjective norms, and perceived behavior control on intentions to engage in pro-environmental behavior [41], and affirmed their predictive role [42]. Similarly, norm activation theory holds that personal norms have a guiding effect on behavior, that is, if an individual believes he has moral obligations to protect the environment, he will take corresponding behaviors [38]. Particularly, this theory emphasizes the activation conditions of personal norms, namely awareness of consequences and ascription of responsibility [43], because inactive norms are difficult to affect individual behavior [44]. 

Other widely used theories include goal-framing theory [45], self-determination theory [46], protective motivation theory [47], stimulus-organism-response paradigm [48], and so on. These theories explain the influence of internal and external factors on individual pro-environmental behavior and provide ideas for understanding the antecedents of this behavior. In addition, psychological theories also provide references for the study of pro-environmental behavior in specific situations. For example, in the organizational context, scholars mainly use social identity theory [49], social exchange theory [50], social learning theory [51], and ability-motivation-opportunity theory [52] to explain the promotion effect of corporate social responsibility, environmental management practice, ethical leadership, and green human resource management on employees’ pro-environmental behavior. In the context of tourism, place attachment theory is used to explain tourists’ environmental responsibility behavior [53,54].

In terms of consequences, identity theory, cognitive dissonance theory, and moral licensing theory are important theoretical bases for spillover effect of pro-environmental behavior [32]. From the perspective of identity, individuals who adopt pro-environment behavior form a kind of self-perception [55]. When individuals see themselves as people who care about the environment, they will continue to behave in ways consistent with this identity, and are more likely to engage in other pro-environment behaviors [56,57]. According to cognitive dissonance theory, individuals are motivated to keep their behavior consistent in order to avoid the inconsistency of knowledge and action. After performing pro-environmental behavior, the desire to show a consistent image urges individuals to engage in other environmental actions [58]. In contrast, according to moral licensing theory, previous pro-environmental behavior improves the individual’s perception of their own moral image and allows them to no longer adopt relevant behaviors [59]. This is a commonly used theory to explain the negative spillover effect of pro-environmental behavior, but it has not been widely supported by data [32]. Therefore, the application of moral licensing theory in the study of pro-environmental behavior needs further research.

#### 4.1.2. Application of Sociological Theories in the Study of Pro-Environmental Behavior

With the development of pro-environmental behavior research, scholars tried to expand their research horizons. Some studies examined pro-environmental behavior based on sociological theories, focusing on the role of social interaction, social capital, and other factors [15]. The introduction of these theories emphasizes the influence of social situational factors on individual behavior, which helps to comprehensively consider the relationship between nature, society, and individuals [60], and further expand the understanding of pro-environmental behavior. As members of a social network, individuals inevitably interact with other members of the network and are influenced by them when making behavioral decisions [61]. Therefore, social interaction theory has become an important theoretical basis to explain how pro-environmental behavior is formed in social situations. Previous studies showed that social interaction provides individuals with useful information [11], and improves their understanding of environmental issues and environmental protection knowledge level, which promotes individuals to perform environmental protection practice [15,62]. At the same time, the social interaction or relationship structure between individuals generates social capital, that is, the potential available resources accumulated in the social network [14,63,64]. Its prediction of pro-environmental behavior is another important contribution of sociological theories to the theoretical development of pro-environmental behavior. By aligning self-interest with collective interest, instilling pro-environmental values, informing of collective need, enabling actions, and establishing norms, social capital promotes individual environmental behavior [65]. What is more, social practice theory provides a perspective focusing on environmental protection practice itself, which is very different from previous views. For pro-environmental behaviors, different forms of behaviors are interrelated and share resources. Therefore, after taking one type of pro-environmental behavior, individuals may adopt other forms of environmental protection practice [66]. Social practice theory has shown unique value in explaining the spillover effect of pro-environment behavior, but the current evidence is not sufficient, which provides clues for future research.

#### 4.1.3. Application of Economics Theories in the Study of Pro-Environmental Behavior

Compared with scholars in psychology and sociology, economists pay more attention to the influence of external factors such as price and income when studying pro-environmental behavior, which provides a more realistic perspective for the research [67]. According to the views of traditional economics and neoclassical economics, people are rational and pursue the maximization of personal utility. Under this analytical logic, economic incentive is an effective way to promote individuals to protect the environment and adopt pro-environmental behavior [68]. Relevant studies mainly use rational choice theory to clarify the above relationship [69]. However, experimental evidence showed that individuals are not always rational when taking actions, and often deviate from the hypothesis of rational choice theory [70]. In this case, the relationship between economic incentives and pro-environmental behavior has been reconsidered, and scholars have extended the rational choice model. A typical example is that the utility function is extended to include various intrinsic motives, that is, motivation crowding theory [69]. It holds that external motivations conflict with other motivations and weaken internal motivations [71], which has an adverse impact on expected behavior in the long run [72]. This theory has insufficient experimental evidence in explaining pro-environmental behavior, and there is still sufficient research potential [73]. In addition, pro-environmental behavior could be regarded as individual efforts for collective environmental quality, that is, private provision of public goods. Under this analytical framework, income plays an important role. Previous research showed that unemployment leads to decrease pro-environmental behavior requiring financial contributions and increase pro-environmental behavior that requires mainly time/effort as inputs [6]. The rebound effect related to energy use is another important research issue which is a concern of economists, but no consistent conclusion has been reached [74]. The relationship between energy efficiency improvement and energy service demand deserves further consideration in the future.

### 4.2. Theoretical Exploration of Pro-Environment Behavior

#### 4.2.1. The Proposition and Development of Value-Belief-Norm Theory

Values and personal norms are important internal factors affecting individual pro-environmental behavior. Early scholars recognized their importance and conducted detailed research [75], but failed to connect them theoretically for systematic analysis and test. In order to coherently consider the drivers of environmentalism and the formation process of pro-environmental behavior, Stern and his colleagues constructed the value-belief-norm theory [76,77]. This theory expands norm activation theory [78] by value- based theory [79] and new environmental paradigm [80], forming a causal chain. Specifically, the causal chain contains five variables that contribute to the generation of pro-environmental behavior: values, new environmental paradigm, awareness of adverse consequences, ascription of responsibility to self and personal pro-environmental norms. Each variable affects the next variable, or directly affects other variables in the chain [8,81]. Compared with other theories at that time, such as cultural theory and the theory of post material values, value-belief-norm theory has the best explanation effect on pro-environmental behavior [77]. Therefore, this theory has been applied to different groups in many studies [9], and has become one of the most important theoretical foundations for subsequent studies on pro-environmental behavior. 

With the deepening of research, value-belief-norm theory has been expanded or adjusted in order to better reflect the actual situation or guide practice. Awareness of consequences may stimulate anticipated pride or guilt [82], and these positive or negative emotions are usually closely related to pro-environmental behavior [83]. Therefore, the emotional process is incorporated into the theoretical model, forming a causal chain of value-belief-emotion-norm. The integration of expected emotion improves the clarity and explanatory power of causal chain, which is of great significance for a fuller understanding of the formation of pro-environmental behavior [84]. In addition, values are relatively stable and difficult to be changed by external factors [85,86], so this section of the theory is of limited use for policy suggestions. Replacing values with environmental knowledge, an external factor affected by environmental education, could improve the practical value of this theory and provide ideas for the government to guide pro-environmental behavior [87].

#### 4.2.2. Behavioral Theories Related to Contexts

The explanation of internal factors on pro-environmental behavior is incomplete [40], and it is necessary to bring external conditions into the theoretical framework. Therefore, contextual factors are taken into account and a series of theoretical models are produced. Among them, attitude-behavior-context theory provides a relatively simple and clear view for pro-environmental behavior [88]. This theory affirms the role of internal factors represented by attitude and external factors represented by context on pro-environmental behavior, and has become a common theoretical basis for follow-up research [89]. In particular, the theory assumes that there is an interactive effect between attitude and contextual factors in influencing pro-environmental behavior, in other words, contextual factors affect the relationship between attitude and behavior. This may provide a theoretical explanation for the attitude-behavior gap of individuals in pro-environmental behavior [90]. In addition, considering the neglect of values and situational context in the theory of reasoned action, a relatively developed conceptual framework has emerged [91]. Relatively developed means that this framework has expanded the theory of reasoned action to some extent. Beside affirming the predictive effect of intention on behavior, it pays attention to the positive effects of environmental values, contextual factors and psychological factors on intention and pro-environmental behavior [92]. Some specific variables are included, such as behavioral context and behavioral specific attributes [93]. Compared with attitude-behavior-context theory, this framework is more abundant and provides insights into the difference between intention and behavior, which has always been concerned in the study of pro-environmental behavior.

Focusing on external conditions provides an alternative framework for pro-environmental behavior. The framework divides these factors into characteristics of local environments, social network characteristics and community characteristics, and explores how these factors and personal characteristics affect personal environmental action tendency and ability, which drives environmental action [94]. This framework enriches the understanding of situational factors and lays a theoretical foundation for subsequent analysis of the role of situational factors on pro-environmental behavior. It is worth noting that online technologies have great potential in promoting pro-environmental behavior [95]. To be specific, through informational, relational and experiential functions, online technologies produce personal, social or situational factors affecting pro-environmental behavior, and promote the transformation of these factors into behavior. Due to the lack of relevant research, there are many questions in this field that deserve further discussion.

#### 4.2.3. Construction and Development of Pro-Environmental Behavior Decision Models

With the development of pro-environmental behavior research, scholars are not satisfied with a one-sided view of pro-environmental behavior from an existing theoretical perspective, and are committed to building a more comprehensive pro-environmental behavior determination model. Through meta-analysis of the existing literature, a series of key variables affecting pro-environmental behavior were identified and a model of responsible environmental behavior was formed [96]. This model pointed out that environmental behavior is affected by situational factors and behavioral intention, which is closely related to cognitive factors represented by knowledge and personal factors represented by attitude [97]. Unlike other theories, this model is a summary of the existing research contents, which fully reflects the known conditions for the formation of pro-environmental behavior at that time. Similarly, drawing on ideas from several existing theories, a comprehensive action decision model was developed [35]. In addition to situation and intention, it assumes that behavior is also determined by habit, and the normative process is the indirect source of behavior. What is more, four factors interact in a complex way and ultimately affect pro-environmental behavior. This model provides a favorable theoretical support for the intervention of pro-environmental behavior [98]. In addition, due to the conflict between different individual goals, reducing pro-environmental costs to defuse the conflict between normative goal and the other two goals, and strengthening normative goals, all become effective ways to encourage pro-environmental behavior [99]. The influence of goals is related to values, which can be activated or deactivated by situational factors. These factors and relationships were combined into an integrated framework for encouraging pro-environmental behavior, indicating the role of different goals and interventions [100], which is of great significance to the formulation of environmental policies.

### 4.3. Theoretical Integration of Pro-Environment Behavior: Future Directions

#### 4.3.1. The Combination of Rationality and Sensibility

Based on economic theories, individuals seek to maximize their utility and act in a highly rational manner. In this case, pro-environmental behavior is usually not the autonomous action of the individual. This is a rational perspective to analyze individual pro-environmental behavior. In fact, people often behave differently from the expectations proposed by these theoretical assumptions [70], because they ignore the complex psychological processes of individuals. Psychological factors such as anticipated guilt and pride experience promote individuals to actively practice pro-environmental behavior [101,102]. This is a perceptual perspective to analyze individual pro-environmental behavior. Relevant research basically uses psychological theories as the theoretical basis. What should be noted is that these theories underestimate the impact of economic costs on decision making to some extent. Since both rational and perceptual perspectives have some emphasis on explaining pro-environmental behavior and could complement each other, the integration between them is the direction that scholars can study in the future. For example, the differential impact of moral emotion on pro-environmental behavior with different costs may be an interesting question. 

Rationality and sensibility can also be explained in another way, that is, rationality corresponds to cognitive factors and sensibility corresponds to emotional factors. Most of the existing theories treat cognitive and emotional factors separately. For example, the theory of planned behavior focuses on cognitive factors such as attitudes, subjective norms, and perceived behavior control [39], while ignoring the role of emotional factors. This leads to the theoretical explanation of practical behavior which is not comprehensive and systematic. Therefore, integrating cognitive and emotional factors to form a pro-environmental behavior theory combining rationality and sensibility becomes an important content of future research.

#### 4.3.2. The Combination of External and Internal Causes

Compared with the external factors, the existing theories and studies pay more attention to the influence of internal factors on individual pro-environmental behavior and the complex psychological process before taking on behavior. The application of various psychological theories in the study and the proposal of value-belief-norm theory proves this view. However, the understanding of pro-environmental behavior from this perspective is limited [40]. Some questions, such as how individuals behave differently in different contexts, have not been answered. The rise of contextual factors and related theories has theoretically affirmed the role of external conditions on pro-environmental behavior [89], emphasized the effectiveness of external factor paths, and contributed to the comprehensive development of research. Therefore, comprehensive consideration of internal and external factors to form an integrated pro-environmental behavior theory is helpful to explain the behavior. Existing research has made some efforts in this regard [87], and more abundant studies can be conducted in the future.

## 5. Discussion

This study reviewed the research status of pro-environmental behavior in terms of literature publication, research hotspots and topics, and summarized its theoretical progress. Through the current and recent status of literature publication, it demonstrates the growing interest of scholars in this research issue, which is consistent with the results of previous studies [3]. This paper presented three important categories of research topics, namely pro-environmental behavior of individuals with different roles, antecedents, and consequences of the behavior. They cover almost all research content and help to position existing studies. Most importantly, paths of theoretical progress were summarized: theoretical development, theoretical exploration, and theoretical integration. This is the gap of existing studies and the main contribution of this study. In terms of theoretical development, psychological theories are most commonly applied in pro-environmental behavior research, especially the theory of planned behavior and norm activation theory. Sociological and economic theories have been applied relatively less frequently. This path emphasizes the application of existing theories, so contributes little to the development of theories. Theoretical exploration emphasizes the extension and innovation of theories, which contributes more than theoretical development and provides the theoretical basis for subsequent research. For example, value-belief-norm theory is subsequently often applied to pro-environmental behavior studies [9]. Since a single theory is limited in explaining pro-environmental behavior, theoretical integration is the future development direction of theory. It helps to develop a more comprehensive perspective and an integrated view of pro-environmental behavior.

## 6. Conclusions

This paper comprehensively showed the research overview of pro-environmental behavior, and clearly expounded the theoretical progress of pro-environmental behavior, which is conducive to accelerating the subsequent scholars’ mastery of corresponding knowledge and promoting the development of pro-environmental behavior research and theory. At the same time, there are some limitations in this study. Firstly, this study focused on individuals’ pro-environmental behavior and did not consider this behavior of subjects at other levels such as enterprises. These have differences in manifestations, influencing factors and outcome variables, but these differences were not reflected in this study, which affects the applicability of the study to some extent. Secondly, this study collected more than 40 years of literature data for analysis, but did not compare the contents of professional journals of environmental behavior with those of other journals, which failed to clarify the similarities and differences between the topics they focused on. Finally, pro-environmental behavior is a multidisciplinary issue. Although theories of many disciplines were mentioned in this paper, the key points and concerns of each theory were not deeply analyzed, which needs to be supplemented by follow-up research.

It is proposed that theoretical integration is the future direction of theoretical progress, implying that the study of pro-environmental behavior from the perspective of combining rationality and sensibility or exogenous and endogenous factors will become an important research content. In addition, according to the results of the literature review, the application of moral licensing theory, social practice theory, and motivation crowding theory in the study of spillover effects or formation mechanisms of pro-environmental behaviors has not been widely supported by data, and further and detailed studies are needed in the future.

## Figures and Tables

**Table 1:** Co-occurrence analysis results (Top 10 keywords).

Keyword	Frequency	Year	Centrality
attitude	471	1995	0.03
pro-environmental behavior	450	2005	0.04
planned behavior	334	2003	0.02
determinant	278	2004	0.03
value	256	1998	0.02
intention	251	1995	0.03
model	220	1995	0.06
consumption	171	1991	0.03
climate change	153	2013	0.01
knowledge	138	1998	0.03

**Table 2:** Cluster analysis results (Top 10 clusters).

Number	Size	Silhouette	Mean (Year)	Top Terms (LLR)
#1	87	0.717	2014	corporate social responsibility; organizational identification; green human resource management; employee green behavior
#2	77	0.774	2010	environmentally responsible behavior; place attachment; ecological behavior; nature-based tourism
#3	71	0.765	2013	sustainable consumption behavior; urban residents; impact; energy-saving behavior
#4	71	0.706	2010	theory of planned behavior; habit; personal norm; perceived behavioral control
#5	67	0.881	2013	green intention; triple bottom line; quantitative methods; renewable energy
#6	66	0.767	2012	knowledge; university students; higher education; sustainable consumption
#7	64	0.715	2006	value; attitude; belief; pro-environmental behavior
#8	62	0.804	2009	spillover; social norms; efficiency; psychological distance
#9	60	0.676	2009	moral licensing; future orientation; time perspective; psychology
#10	51	0.808	2011	social identity; public goods; activism; happiness; prosocial behavior

**Table 3:** Summary of theories.

Path	Classification	Theory
theoretical development	psychological theory	theory of planned behavior
		norm activation theory
		goal-framing theory
		self-determination theory
		protective motivation theory
		stimulus-organism-response paradigm
		social identity theory
		social exchange theory
		social learning theory
		ability-motivation-opportunity theory
		place attachment theory
		identity theory
		cognitive dissonance theory
		moral licensing theory
	sociological theory	social interaction theory
		social practice theory
	economics theory	rational choice theory
		motivation crowding theory
		private provision of public goods
		rebound effect
theoretical exploration	value-belief-norm theory	value-belief-norm theory
	behavioral theories related to contexts	attitude-behavior-context theory
		conceptual framework of environmental behavior
		conceptual framework of environmental action
	pro-environmental behavior decision model	model of responsible environmental behavior
		comprehensive action decision model
		integrated framework for encouraging pro-environmental behavior
theoretical integration	the combination of rationality and sensibility	
	the combination of external and internal causes	

## Data Availability

Not applicable.

## Supplementary Materials

The following supporting information can be downloaded at: https://www.mdpi.com/article/10.3390/ijerph19116721/s1, Table S1: A list of all the papers used in the study.
